# Obesity and atypical depression symptoms: findings from Mendelian randomization in two European cohorts

**DOI:** 10.1038/s41398-021-01236-7

**Published:** 2021-02-04

**Authors:** Giorgio Pistis, Yuri Milaneschi, Caroline L. Vandeleur, Aurélie M. Lasserre, Brenda W.J.H. Penninx, Femke Lamers, Dorret I. Boomsma, Jouke-Jan Hottenga, Pedro Marques-Vidal, Peter Vollenweider, Gérard Waeber, Jean-Michel Aubry, Martin Preisig, Zoltán Kutalik

**Affiliations:** 1 Department of Psychiatry, Lausanne University Hospital and University of Lausanne, Lausanne, Switzerland; 2 Department of Psychiatry, Amsterdam Public Health and Amsterdam Neuroscience, Vrije Universiteit Medical Center and GGZ inGeest, Amsterdam, The Netherlands; 3 Department of Biological Psychology, Vrije Universiteit, Amsterdam, The Netherlands; 4 Department of Medicine, Lausanne University Hospital and University of Lausanne, Lausanne, Switzerland; 5 Department of Psychiatry, University Hospital of Geneva, Geneva, Switzerland; 6 Institute of Primary Care and Public Health (Unisante), University of Lausanne, Lausanne, Switzerland; 7 Swiss Institute of Bioinformatics, Lausanne, Switzerland

**Keywords:** Personalized medicine, Depression

## Abstract

Studies considering the causal role of body mass index (BMI) for the predisposition of major depressive disorder (MDD) based on a Mendelian Randomization (MR) approach have shown contradictory results. These inconsistent findings may be attributable to the heterogeneity of MDD; in fact, several studies have documented associations between BMI and mainly the atypical subtype of MDD. Using a MR approach, we investigated the potential causal role of obesity in both the atypical subtype and its five specific symptoms assessed according to the Statistical Manual of Mental Disorders (DSM), in two large European cohorts, CoLaus|PsyCoLaus (*n* = 3350, 1461 cases and 1889 controls) and NESDA|NTR (*n* = 4139, 1182 cases and 2957 controls). We first tested general obesity measured by BMI and then the body fat distribution measured by waist-to-hip ratio (WHR). Results suggested that BMI is potentially causally related to the symptom increase in appetite, for which inverse variance weighted, simple median and weighted median MR regression estimated slopes were 0.68 (SE = 0.23, *p* = 0.004), 0.77 (SE = 0.37, *p* = 0.036), and 1.11 (SE = 0.39, *p* = 0.004). No causal effect of BMI or WHR was found on the risk of the atypical subtype or for any of the other atypical symptoms. Our findings show that higher obesity is likely causal for the specific symptom of increase in appetite in depressed participants and reiterate the need to study depression at the granular level of its symptoms to further elucidate potential causal relationships and gain additional insight into its biological underpinnings.

## Introduction

Major depressive disorder (MDD) and obesity are major public health concerns worldwide, which entail an excess of premature mortality^1,2^. Studies have repeatedly documented a strong association between depressive disorders and obesity^3–7^, but the mechanisms underlying this association are still poorly understood^8,9^. Over the last years, clinical heterogeneity has represented a challenging impediment to unraveling the complex biology of MDD and to the comprehension of its relationship with obesity^9^. The characterization of MDD subtypes, and the consequent disentanglement of the heterogeneity of depression, represented a promising strategy to gain more insight into biological mechanisms related to MDD^8,10^. Indeed, using this approach several studies documented associations between depression and obesity markers that were mainly restricted to patients endorsing a symptom profile that has often been labeled as “atypical”^11–17^. According to the fourth edition of the Diagnostic and Statistical Manual of Mental Disorders (DSM-IV)^18^ specifier, atypical depression is defined by the presence of mood reactivity and two of the following: hypersomnia, leaden paralysis, increase in appetite/weight gain, and long-standing pattern of interpersonal rejection sensitivity. Nevertheless, previous studies applied the label of “atypical” to subgroups of patients selected according to different definitions: while some studies^19,20^ strictly applied the DSM criteria for the atypical specifier, others^13,14^ used a simplified definition based on few symptoms (e.g. increase in appetite, hypersomnia, fatigue), or used classifications based on data-driven methods^21^. ﻿It is crucial therefore to identify which of the atypical symptoms may constitute a major driver of the associations with obesity. A recent review^22^ showed indeed that obesity-related immuno-metabolic dysregulations map more consistently to atypical, energy-related symptoms (e.g. hypersomnia, increase in appetite, leaden paralysis, and fatigue) rather than the entire classical construct of atypical depression as coded in the DSM. A multicenter study of 14 datasets of the Psychiatric Genomics Consortium (PGC) recently assessed participants with MDD and found a strong positive genetic correlation between MDD patients with increased appetite or weight and body mass index (BMI), whereas MDD patients with decreased appetite or weight revealed an inverse genetic correlation with BMI^9^. Another recent study^23^ found that MDD patients endorsing weight gain and hypersomnia carried a higher genetic loading for BMI than those without these symptoms. The association between obesity and atypical depressive symptoms could be either causal or attributable to shared etiological factors such as genes. Prospective clinical and epidemiological studies suggested that an elevated BMI predisposes to atypical-like depressive syndromes^24,25^ but also the converse^11,15^. Recently, the Mendelian Randomization (MR) method was applied to examine whether the relationship between obesity and the overall diagnosis of depression is likely to be causal. MR is a potent tool which uses exposure-associated genetic variants as instrumental variables to estimate the causal effect of this exposure on an outcome of interest^26^. The association between a genetic variation and an outcome is generally not susceptible to reverse causation or confounding that may distort interpretations of conventional observational studies^27^. Studies using the MR approach showed evidence for a causal role of depression on BMI increase^28^, and revealed conflicting results regarding the causal role of high BMI in increasing symptom severity scores or the diagnosis of depression^29–35^. In particular, despite the large overlap of included studies, the two largest genome-wide association study (GWAS) meta-analyses on depression by Wray et al.^34^ and Howard et al.^35^ suggested causal and non-causal effects of BMI on depression, respectively. Another recent study using the MR approach found that higher BMI could be a causal risk factor for, among other depressive symptoms, changes in appetite but could not differentiate between the underlying diametrically opposite symptoms of increase and decrease in appetite^36^. Again, these inconsistent findings are likely to be attributable to the heterogeneity of depression involving the mixing of the liabilities of different MDD subtypes together and emphasize the importance of investigating whether the causal association between obesity and depression is restricted to specific symptom profiles. The aims of the present study were to test a potential causal role of obesity on both the atypical MDD subtype and its five specific symptoms, assessed according to the DSM-IV, in two large cohorts. Applying several kinds of MR methods, we primarily tested the BMI as a measure of general obesity. In a secondary analysis, we tested the body fat distribution, measured by waist-to-hip ratio (WHR).

## Materials and methods

The analyses were conducted in two cohort studies, the CoLaus|PsyCoLaus and the NESDA|NTR cohorts.

### Participants

#### CoLaus*|*PsyCoLaus

CoLaus|PsyCoLaus^19,37^ is a prospective cohort study with a baseline and two follow-up assessments to date designed to study mental disorders and cardiovascular risk factors in the community and to determine their associations. The sample of 6734 participants was randomly selected from the 35- to 75-year-old residents of the city of Lausanne (Switzerland) from 2003 to 2006 according to the civil register. Full genetic data were only available for Caucasians. The final sample comprised 3350 participants from 35 to 67 years and for whom combined genetic and psychiatric data were available. From the final sample of CoLaus|PsyCoLaus participants, 53.31% were females, the mean age was 51.8 years (SD 8.63 years) and the mean year of birth was 1954 (SD 8.60 years). Diagnostic information on mental disorders was collected at baseline using the French version^20^ of the semi-structured Diagnostic Interview for Genetic Studies (DIGS)^38^. Lifetime psychiatric diagnoses were assigned according to the fourth edition of the DSM-IV (ref. ^18^). The French translation of the DIGS revealed excellent inter-rater (Kappa = 0.93) and fair-to-good test–retest reliability (Kappa = 0.62) for lifetime diagnoses of MDD^20^. This interview, which includes questions on increase in appetite or significant weight gain and hypersomnia, was completed with additional questions on mood reactivity, leaden paralysis, and interpersonal rejection sensitivity in order to elicit all five criteria of the specifier for atypical depression features according to DSM-IV. The interview systematically assesses the last and the most severe depressive episodes. A diagnosis of atypical MDD was assigned if criteria for atypical features were met according to the specifier in at least one of the two described episodes. The DSM-IV criteria for atypical MDD include (1) mood reactivity and at least two of the following four symptoms: (2) increase in appetite or significant weight gain, (3) hypersomnia, (4) leaden paralysis, and (5) interpersonal rejection sensitivity. The DIGS also collects data on a series of socio-demographic factors including year of birth and gender, which were controlled for in all analyses. To avoid potential redundancy with obesity measures, we only applied the appetite part of the appetite/weight-gain criterion. Interviewers were required to be masters-level psychologists, and were trained over a 1- to 2-month period. During data collection, each interview was reviewed by an experienced senior clinical psychologist. The control group included participants who did not meet criteria for MDD, but also not for bipolar disorder, schizoaffective disorder, or schizophrenia. The Institutional Ethics’ Committee of the University of Lausanne approved the CoLaus|PsyCoLaus study. All participants signed a written informed consent after having received a detailed description of the goal and funding of the study.

Body weight, height, waist and hip circumferences were measured at the out-patient clinic at the Centre Hospitalier Universitaire Vaudois (CHUV)^37^.

Genotyping, quality control, and imputation are described in Supplementary Methods 1.1.

#### NESDA*|*NTR

NESDA|NTR included 4821 unrelated participants of North-European ancestry from the Netherlands Study of Depression and Anxiety (NESDA, *n* = 2047) and from the Netherlands Twin Register (NTR, *n* = 2774). Detailed descriptions of the rationale, design, and methods for both studies are given elsewhere^21,39^. Briefly, NESDA is an ongoing cohort study on the long-term course and consequences of depressive and anxiety disorders. In 2004–2007, 2981 participants with depression and anxiety disorders and healthy controls aged 18–65 years were recruited from the community (19%), general practice (54%), and secondary mental health care (27%) and were followed-up during five biannual assessments. The NTR study has been collecting longitudinal data on Dutch adult twin families since 1991 (ref. ^40^). The research protocols from both studies were approved by the Central Ethics Committee on Research Involving Human Subjects of the VU University Medical Center, Amsterdam, an Institutional Review Board certified by the U.S. Office of Human Research Protections (IRB number IRB00002991 under Federal-wide Assurance FWA00017598; IRB/institute codes) and informed consent was obtained from all participants. People with MDD were derived from NESDA. Lifetime MDD diagnoses according to the DSM-IV and specific atypical symptoms were ascertained using the Composite Interview Diagnostic Instrument, which at the 9-year follow-up was extended with additional questions in order to elicit all atypical symptoms^41^. Healthy controls were screened based on the absence of any lifetime psychiatric disorder (NESDA). NTR controls were selected based on no report of MDD, no known first-degree relatives with MDD, and a low factor score based on a multivariate analysis of depressive complaints, anxiety, neuroticism, and somatic anxiety^42^.

Body weight, height, waist and hip circumferences were measured by medical examination at the study clinic during the visit for NESDA^21^ and during the home visit after blood sampling for NTR^43^.

Genotyping, quality control, and imputation are described in Supplementary Methods 1.2.

### Statistical analyses

We tested BMI and WHR as measures of obesity (exposures) and used two-sample MR^44,45^ to test whether they are causal risk factors for six different outcomes. One outcome was based on the MDD atypical subtype definition: (1) atypicals vs controls, and (2–6) the presence or absence of each of the five atypical subtype symptoms. MR infers causality by using genetic variants (as instrumental variables) reliably associated with an exposure and regressing their effect on the exposure against their effect on the outcome. Summary statistics for BMI^46^ and WHR^47^ were obtained from large GWAS of international consortia and genome-wide significant independent SNPs were selected as instruments (Supplementary Methods 2.1). An important assumption of MR is that each SNP must only influence the risk of the outcome through the exposure under investigation, as the inclusion of SNPs that contribute through a pleiotropic pathway could bias estimates^48^. To assess for the presence of directional horizontal pleiotropy, we used MR-Egger regression^49^. We performed inverse variance weighted (IVW) instrumental variable analysis followed by sensitivity analyses based on weighted-median and simple-median causal estimators, which provide consistent causal estimates even when up to half of instruments are violating MR assumptions. Analyses were conducted using the TwoSampleMR R package^44,45^ (Supplementary Methods 2.2). Since population stratification is another potential source of bias for MR analyses, we selected summary statistics from GWAS that included only individuals of European descent for both BMI and WHR. CoLaus|PsyCoLaus and NESDA|NTR were two cohorts which contributed to both the BMI and WHR meta-analyses, together providing respectively 8924 and 8914 individuals overall and accounting for 1.31% and 1.28% of the two meta-analysis sample sizes. There are two kinds of biases present in the two-sample MR context: overlapping samples lead to bias toward the observational correlation^50,51^, while winner’s course biases the causal effect estimate toward zero. Both biases are mitigated by the use of strong instruments. This was confirmed in CoLaus|PsyCoLaus given that the instrumental variables for BMI and WHR revealed strong associations with their respective phenotypes (*F* = 30.74 for BMI and *F* = 13.45 for WHR). Usually *F* > 10.0 is required for an adequate instrumental variable^26^. The two instrumental variables explained 5.5% and 2.4% of the phenotypic variance, respectively. For each outcome, we conducted a case-control GWAS separately on CoLaus|PsyCoLaus and NESDA|NTR and then we ran fixed effects meta-analyses of the results of the two cohorts using the IVW method implemented in METAL^52^ (Supplementary Tables 1–14). Logistic regression models were adjusted for year of birth, gender, and the first five ancestry-informative genetic principal components. Models on symptoms were also additionally adjusted for the other four symptoms. For the analyses involving the five atypical symptoms, the significance level was set to *p* = 0.01 (0.05/5) according to the Bonferroni correction for multiple testing. In NESDA|NTR, these analyses were based on the whole sample with the coding of depressive symptoms as absent in controls, whereas in CoLaus|PsyCoLaus the respective analyses were restricted to participants who entered the depression section.

## Results

### MDD atypical subtype and atypical symptoms in the two cohorts

Table 1 provides the description of the 2643 participants with lifetime MDD and the 4846 controls of the two cohorts. Among the participants with MDD, 565 met criteria for atypical MDD according to the DSM-IV. As expected, the age of onset of MDD was lower and the number of episodes was higher in the clinical NESDA|NTR study as compared to the population-based CoLaus|PsyColaus study.

**Table 1:** Demographic and clinical characteristics and distribution of atypical symptoms in CoLaus|PsyCoLaus and NESDA|NTR.

	CoLaus|PsyCoLaus				NESDA | NTR			
	MDD	Atypical MDD	No MDD	Participants endorsing atypical symptoms	MDD	Atypical MDD	No MDD	Participants endorsing atypical symptoms
Total *N*	1461	386	1889	1958	1182	179	2957	4139
Female (%)	66.12	72.54	43.41	63.02	68.52	70.39	60.97	63.13
Mean year of birth (SD)	1955 (8.5)	1955 (8.3)	1954 (8.7)	1955 (8.5)	1962 (12.5)	1963 (12.1)	1963 (15.7)	1963 (14.9)
*Number of episodes (%):*								
1 episode	59.07	48.70	–	–	1.70	1.60	–	–
2 episodes	22.38	25.91	–	–	22.60	16.10	–	–
>2 episodes	18.48	25.39	–	–	75.70	82.30	–	–
Mean age of onset (SD)	33.81 (12.9)	33.10 (13.4)	–	–	27.8 (12.9)	24.3 (12.4)	–	–
Increase in appetite (%)	17.18	40.67	–	14.15	8.79	37.43	–	2.51
Hypersomnia (%)	24.84	48.70	–	20.38	15.31	56.98	–	4.37
Mood reactivity (%)	69.95	100.00	–	72.37	32.82	100.00	–	9.37
Leaden paralysis (%)	29.77	59.06	–	24.41	23.60	81.56	–	6.74
Rejection sensitivity (%)	66.11	90.15	–	61.08	19.96	75.42	–	5.70

Number of episodes available in 59% of NESDA|NTR MDD cases and 69% of NESDA|NTR atypical MDD cases.

The table also shows the distribution of depressive symptoms in the two cohorts. In CoLaus|PsyCoLaus, the presence of depressive symptoms was assessed in the 1958 participants (i.e. 58.4% of the entire sample) who had entered the depression section (regardless of whether they fulfilled criteria for MDD or not), whereas in NESDA|NTR information on symptoms was only available for participants meeting criteria for MDD (symptoms were recorded as absent for controls).

### MR analyses

**Fig. 1:**
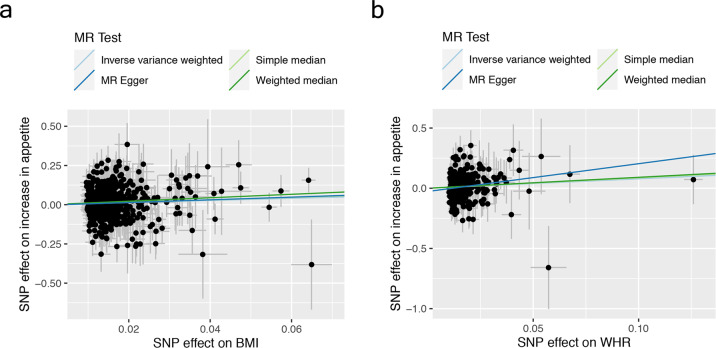
Test of whether BMI and WHR adiposity measures are causal risk factors for increase in appetite. The panels plot per-allele effect sizes for **a** BMI, **b** WHR (*x*-axes) against per-allele effect size for increase in appetite (*y*-axis). For adiposity measures, effect sizes are measured in SDs, for increase in appetite symptom the effect size is log-odds ratio. For each plot, we estimated the slope using inverse-variance weighted regression (light blue line), simple-median regression (light green line), weighted-median regression (dark green line), and Egger regression (dark blue line).

Table 2 and Fig. 1 provide the results from two-sample MR analyses. The analysis testing the effect of BMI on atypical MDD did not reveal evidence for a causal role in the meta-analysis as well as in the two studies. As expected, we did not find evidence for a causal effect of BMI on non-atypical MDD either. Analyses focusing on the atypical symptoms consistently supported an association between the instrumental variable for BMI and the symptom increase in appetite according to all three approaches (IVW *p* = 0.004, simple median *p* = 0.036, and weighted median *p* = 0.004). The IVW and weighted median MR regression estimates remained significant after Bonferroni correction. In contrast, there was no evidence for a causal association between the BMI and atypical symptoms other than increase of appetite. Comparison of the results according to the IVW method in the two studies reveals that the association between BMI and increase in appetite was stronger in CoLaus|PsyCoLaus than in NESDA|NTR. For the other symptoms, CoLaus|PsyCoLaus revealed no associations according to the IVW method, whereas NESDA|NTR even showed significantly negative associations between BMI and mood reactivity as well as rejection sensitivity.

**Table 2:** Results of the Mendelian Randomization analyses in CoLaus|PsyCoLaus, NESDA|NTR, and in their combined dataset.

Dataset	Exposures	Outcomes	*N* SNPs	Inverse variance weighted			Simple median			Weighted median		
				Beta	SE	*p*	Beta	SE	*p*	Beta	SE	*p*
CoLaus|PsyCoLaus + NESDA|NTR combined	BMI	Atypical MDD vs controls	594	−0.14	0.19	0.471	−0.14	0.29	0.632	−0.06	0.30	0.843
		Non-atypical MDD vs controls	593	0.04	0.12	0.731	−0.03	0.18	0.882	−0.07	0.19	0.725
		Increase in appetite	593	**0.68**	**0.23**	0.004*	**0.77**	**0.37**	**0.036**	**1.11**	**0.39**	0.004*
		Hypersomnia	593	0.05	0.20	0.812	0.16	0.29	0.584	0.36	0.34	0.298
		Mood reactivity	593	−0.23	0.16	0.132	−0.36	0.24	0.130	−0.04	0.24	0.881
		Leaden paralysis	593	−0.11	0.17	0.518	−0.21	0.27	0.436	−0.46	0.28	0.096
		Rejection sensitivity	593	−0.23	0.16	0.152	−0.30	0.26	0.248	0.14	0.29	0.643
	WHR	Atypical MDD vs controls	330	0.33	0.25	0.199	−0.09	0.40	0.822	−0.06	0.39	0.877
		Non-atypical MDD vs controls	329	−0.11	0.16	0.482	0.09	0.23	0.691	−0.03	0.24	0.900
		Increase in appetite	330	**0.78**	**0.31**	**0.011**	0.87	0.44	0.050	0.91	0.52	0.078
		Hypersomnia	331	0.46	0.27	0.092	0.64	0.39	0.099	0.32	0.43	0.459
		Mood reactivity	331	0.40	0.22	0.068	0.44	0.30	0.152	0.44	0.32	0.172
		Leaden paralysis	331	0.08	0.22	0.724	0.38	0.34	0.261	0.41	0.34	0.223
		Rejection sensitivity	330	−0.13	0.23	0.568	−0.46	0.31	0.140	−0.09	0.35	0.787
CoLaus|PsyCoLaus	BMI	Atypical MDD vs controls	593	0.24	0.24	0.319	0.30	0.36	0.396	0.26	0.40	0.526
		Non-atypical MDD vs controls	593	−0.13	0.17	0.447	0.13	0.26	0.605	0.19	0.30	0.537
		Increase in appetite	592	**0.82**	**0.28**	0.004*	0.55	0.44	0.206	0.75	0.47	0.113
		Hypersomnia	592	0.35	0.25	0.153	0.19	0.38	0.621	0.47	0.42	0.267
		Mood reactivity	592	0.16	0.21	0.442	0.01	0.33	0.980	**0.74**	**0.35**	**0.036**
		Leaden paralysis	592	0.26	0.24	0.286	−0.02	0.37	0.957	−0.18	0.36	0.607
		Rejection sensitivity	591	0.08	0.20	0.677	−0.05	0.31	0.879	0.02	0.34	0.943
	WHR	Atypical MDD vs controls	330	0.05	0.32	0.879	0.13	0.47	0.781	0.20	0.49	0.678
		Non-atypical MDD vs controls	330	−0.20	0.22	0.352	−0.04	0.35	0.911	−0.14	0.34	0.688
		Increase in appetite	330	0.56	0.40	0.154	0.35	0.56	0.529	0.36	0.58	0.541
		Hypersomnia	330	0.23	0.33	0.487	0.70	0.50	0.164	−0.15	0.50	0.766
		Mood reactivity	330	0.05	0.30	0.861	−0.01	0.43	0.974	−0.11	0.46	0.809
		Leaden paralysis	330	−0.03	0.31	0.935	−0.25	0.45	0.585	−0.43	0.47	0.368
		Rejection sensitivity	330	−0.30	0.27	0.268	−0.62	0.39	0.109	−0.54	0.42	0.194
NESDA|NTR	BMI	Atypical MDD vs controls	592	**−0.80**	**0.32**	**0.013**	**−1.03**	**0.48**	**0.031**	−0.15	0.52	0.771
		Non-atypical MDD vs controls	592	0.19	0.16	0.236	0.23	0.23	0.315	0.38	0.26	0.140
		Increase in appetite	592	0.36	0.42	0.392	0.14	0.62	0.825	0.14	0.73	0.846
		Hypersomnia	592	−0.49	0.32	0.131	−0.45	0.50	0.376	−0.18	0.56	0.742
		Mood reactivity	591	**−0.66**	**0.22**	0.003*	−0.54	0.33	0.103	−0.50	0.37	0.169
		Leaden paralysis	592	**−0.57**	**0.26**	**0.027**	−0.25	0.40	0.541	−0.51	0.44	0.249
		Rejection sensitivity	592	**−0.91**	**0.28**	0.001*	**−0.97**	**0.43**	**0.022**	**−1.11**	**0.49**	**0.023**
	WHR	Atypical MDD vs controls	330	0.81	0.43	0.061	0.22	0.65	0.732	0.84	0.65	0.197
		Non-atypical MDD vs controls	330	0.01	0.22	0.977	0.10	0.31	0.742	−0.09	0.34	0.801
		Increase in appetite	330	**1.37**	**0.53**	0.009*	1.07	0.79	0.172	1.53	0.83	0.066
		Hypersomnia	330	0.82	0.43	0.055	**1.45**	**0.63**	**0.021**	**1.47**	**0.68**	**0.029**
		Mood reactivity	330	**0.77**	**0.32**	**0.017**	0.76	0.45	0.094	0.79	0.48	0.100
		Leaden paralysis	330	0.23	0.35	0.508	−0.15	0.53	0.771	0.01	0.56	0.988
		Rejection sensitivity	329	0.06	0.37	0.877	−0.13	0.56	0.817	0.09	0.60	0.878

Nominal significant associations are in bold and associations on symptoms surviving correction for multiple testing alpha level of 0.01 (0.05/5) are denoted with an asterisk.

*BMI* body mass index, *WHR* waist-to-hip ratio.

Secondary analyses tested the potential causal role of obesity on atypical MDD and its symptoms using WHR as a measure of obesity. This measure did not provide evidence for a causal role of obesity on atypical MDD either. Analyses focusing on the atypical symptoms provided MR estimates that were directionally consistent with those based on BMI. However, the associations between the instrumental variable and the symptom increase in appetite failed to reach the level of significance after Bonferroni correction. Comparison of the results according to the IVW method between the two studies showed a significant association between WHR and increase in appetite in NESDA|NTR but not in CoLaus|PsyCoLaus. Table 3 reveals that the Egger method did not provide evidence for any pleiotropy.

**Table 3:** Results of the Mendelian Randomization analyses using the MR-Egger method in CoLaus|PsyCoLaus, NESDA|NTR, and in their combined dataset.

Dataset	Exposures	Outcomes	*N* SNPs	MR-Egger					
				Intercept			Slope		
				Beta	SE	*p*	Beta	SE	*p*
CoLaus|PsyCoLaus + NESDA|NTR combined	BMI	Atypical MDD vs controls	594	0.002	0.009	0.850	−0.236	0.547	0.666
		Non atypical MDD vs controls	593	−9E−05	0.005	0.987	0.045	0.329	0.891
		Increase in appetite	593	−0.003	0.011	0.778	0.855	0.660	0.196
		Hypersomnia	593	−0.008	0.009	0.398	0.493	0.563	0.382
		Mood reactivity	593	−0.013	0.007	0.064	0.527	0.439	0.231
		Leaden paralysis	593	0.011	0.008	0.169	−0.732	0.482	0.130
		Rejection sensitivity	593	−0.004	0.007	0.545	0.028	0.459	0.952
	WHR	Atypical MDD vs controls	330	−0.019	0.012	0.117	1.388	0.721	0.055
		Non atypical MDD vs controls	329	0.008	0.008	0.275	−0.586	0.462	0.206
		Increase in appetite	330	−0.027	0.015	0.067	**2.309**	**0.885**	0.009*
		Hypersomnia	331	0.004	0.013	0.750	0.223	0.779	0.775
		Mood reactivity	331	−0.007	0.010	0.521	0.778	0.627	0.216
		Leaden paralysis	331	0.013	0.011	0.218	−0.664	0.641	0.301
		Rejection sensitivity	330	−0.009	0.011	0.395	0.391	0.651	0.548
CoLaus|PsyCoLaus	BMI	Atypical MDD vs controls	593	0.004	0.011	0.692	−0.012	0.690	0.986
		Non atypical MDD vs controls	593	0.009	0.008	0.218	−0.684	0.481	0.155
		Increase in appetite	592	0.003	0.013	0.831	0.660	0.793	0.405
		Hypersomnia	592	−0.011	0.011	0.330	0.985	0.694	0.156
		Mood reactivity	592	**−0.022**	**0.010**	**0.024**	**1.448**	**0.605**	**0.017**
		Leaden paralysis	592	0.009	0.011	0.406	−0.274	0.687	0.690
		Rejection sensitivity	591	−0.004	0.009	0.651	0.319	0.559	0.568
	WHR	Atypical MDD vs controls	330	−0.017	0.016	0.277	1.012	0.940	0.283
		Non atypical MDD vs controls	330	0.017	0.011	0.117	−1.161	0.648	0.074
		Increase in appetite	330	−0.035	0.020	0.081	**2.540**	**1.195**	**0.034**
		Hypersomnia	330	0.018	0.016	0.283	−0.769	0.986	0.436
		Mood reactivity	330	−0.008	0.015	0.575	0.520	0.886	0.558
		Leaden paralysis	330	0.019	0.015	0.203	−1.126	0.917	0.220
		Rejection sensitivity	330	−0.014	0.013	0.296	0.485	0.793	0.541
NESDA|NTR	BMI	Atypical MDD vs controls	592	−0.004	0.015	0.808	−0.597	0.914	0.514
		Non atypical MDD vs controls	592	−0.008	0.007	0.256	0.656	0.443	0.139
		Increase in appetite	592	−0.018	0.019	0.349	1.402	1.187	0.238
		Hypersomnia	592	−0.002	0.015	0.886	−0.365	0.915	0.690
		Mood reactivity	591	−0.005	0.010	0.638	−0.384	0.623	0.538
		Leaden paralysis	592	0.013	0.012	0.275	−1.310	0.727	0.072
		Rejection sensitivity	592	−0.005	0.013	0.691	−0.620	0.786	0.431
	WHR	Atypical MDD vs controls	330	−0.018	0.020	0.353	1.825	1.171	0.120
		Non atypical MDD vs controls	330	0.005	0.011	0.627	−0.282	0.633	0.656
		Increase in appetite	330	−0.014	0.024	0.553	2.158	1.422	0.130
		Hypersomnia	330	−0.013	0.020	0.514	1.523	1.160	0.190
		Mood reactivity	330	−0.003	0.015	0.841	0.941	0.894	0.294
		Leaden paralysis	330	0.009	0.016	0.593	−0.250	0.967	0.796
		Rejection sensitivity	329	−0.006	0.017	0.714	0.408	1.025	0.691

Nominal significant associations are in bold and associations on symptoms surviving correction for multiple testing alpha level of 0.01 (0.05/5)) are denoted with an asterisk.

*BMI* body mass index, *WHR* waist-to-hip ratio.

## Discussion

The present paper is, to our knowledge, the first to investigate the causal relationship between obesity and the MDD atypical subtype and its specific symptoms according to the DSM specifier using a MR approach. We tested BMI and WHR as measures of obesity. Our analyses supported a causal link between BMI and the atypical symptom increase in appetite but not atypical MDD as a whole. In contrast, we did not find evidence of a causal effect of BMI on the other atypical symptoms. The differential results across the five symptoms of atypical MDD suggest genetic heterogeneity, which also accounts for the observed absence of an association between BMI and atypical MDD. Our result, suggesting a causal link between BMI and increase in appetite, is compatible with those from a recent MR study by Kappelmann et al.^36^, which found that higher BMI could be causally related to the symptom change in appetite. However, the authors could not differentiate between the underlying diametrically opposite symptoms of increase and decrease in appetite. In contrast to BMI, the association between WHR and an increase in appetite shortly failed to reach the level of statistical significance according to the IVW method in our study, although the effect size for this association was even higher than that established for the association between BMI and increase in appetite (*β* = 0.78 vs *β* = 0.68, respectively). This apparently paradoxical result is likely to be attributable to the weaker instrumental variable for WHR, which was based on a smaller number SNPs entailing a larger standard error and a weaker association with its phenotype.

Although our results supported a causal relationship between BMI and increase in appetite during depression, which is one of five criteria for atypical depression according to the DSM-IV, we did not find evidence for a causal relationship between BMI and atypical depression as a whole. The latter finding can be explained by the absence of evidence for causal associations between BMI and all atypical symptoms other than increase in appetite, whereas increase in appetite is not even a mandatory criterion for atypical depression. Hence, the BMI is likely to be more strongly causally associated with a depression subtype that is solely characterized by a mandatory increase in appetite rather than with atypical depression according to the DSM-IV definition. The causal effect of BMI over the symptom increase in appetite during depression may find an explanation in the alteration of inflammatory, metabolic, and bioenergetics biological pathways resulting in hyperphagia, which is central for obesity and can also occur during depressive episodes. This hypothesis would be compatible with results of a previous study demonstrating positive associations between the polygenic risk scores for BMI, leptin, and C-reactive protein and a major depression subtype characterized by increase in appetite or weight^9^. In contrast, according to our results, the frequently observed association between BMI and atypical depression^11–17^ cannot be explained by a relationship with BMI causing this depression subtype. An inverse causal relationship or shared genetic liability could account for the phenotypic association between BMI and atypical depression. Indeed, prospective data revealed that atypical depression is a risk factor for a subsequent increase in obesity markers^11,15^. Unfortunately, we lack a polygenic risk score for atypical depression, which would allow us to test causality using the MR approach. Previous results of the NESDA study have also supported shared genetic liability between BMI and atypical depression. Indeed, a polymorphism of the *FTO* gene, which has been shown to be consistently associated with obesity and body mass regulation in Europeans^53–56^, was also found to be positively associated with atypical depression^57^.

### Limitations

The results of the present study need to be viewed in the light of several limitations. First, the two cohorts relied on partially different methodologies regarding the sampling of cases and controls as well as the instruments used for diagnostic assessment. The differential methodology is likely to account for heterogeneity between the two samples, which is reflected by differential depression severity and distributions of atypical symptoms. Second, we could not test for a potential bidirectional causal relationship between atypical MDD or its symptoms and BMI or WHR, because large and sufficiently powered GWAS meta-analyses performed on the atypical depression subtype or atypical symptoms are still lacking. Therefore, at this stage there are no instrumental variables available for atypical MDD or atypical depression symptoms which would allow us to test for causal effects of atypical MDD or atypical symptoms on BMI or WHR according to the two-sample MR approach. Moreover, a one-sample MR analysis could not be conducted in the CoLaus|PsyCoLaus and NESDA|NTR cohorts given an insufficient sample size. This may be done in the future when robust GWAS on atypical depression and on its symptoms will become available.

Third, limited statistical power due to limited sample size in the two studies could account for negative findings. Indeed, beside the association between WHR and increase in appetite, which shortly failed to reach the level of statistical significance, also the associations between WHR and both hypersomnia and mood reactivity may have reached statistical significance in larger samples according to the IVW method.

## Conclusions

In the last years, the heterogeneity of depression has been partially disentangled by the characterization of its subtypes which represented a promising strategy to gain additional insight into its complex pathophysiology and relationship with obesity. Recent evidence converges in indicating that immuno-metabolic biological dysregulations map more consistently to atypical symptom profiles characterized by alterations in energy homeostasis (increase in appetite, hypersomnia, leaden paralysis, fatigue)^22^. Building on this evidence, the existence of an “immune-metabolic depression” (IMD) dimension emerging from the clustering of energy-related clinical symptoms with inflammatory and metabolic dysregulations has been postulated. Findings from the present study partially support this hypothesis, showing that the liability for higher BMI is potentially causally related with the development of the specific symptom of increase in appetite in depressed participants. Further experimental and functional studies will be needed to fully elucidate the mechanistic chains underlying this causal association. Overall, results from our analyses reiterate the need to study depression at the granular level of its individual symptoms to further elucidate potential causal relationships and gain additional insight into the biological mechanisms of depression.

## Supplementary information

SUPPLEMENTAL MATERIAL

Dataset 1
