# Supplementary material for: Obesity and atypical depression symptoms: findings from Mendelian randomization in two European cohorts
Source: Transl Psychiatry. 2021 Feb 4;11:96. doi: 10.1038/s41398-021-01236-7 (PMC7862438; doi:10.1038/s41398-021-01236-7)
Supplement: Supplementary file 1 — SUPPLEMENTAL MATERIAL [file 41398_2021_1236_MOESM1_ESM.docx]

**SUPPLEMENTARY METHODS**

***1. Genotyping, quality control and imputation***

***1.1 CoLaus|PsyCoLaus***

***1.2 NESDA|NTR***

***2. Mendelian Randomization (MR)***

***2.1 Selection of independent SNPs as instruments for MR***

***2.2 MR analyses***

***References***

***1. Genotyping, quality control and imputation***

***1.1 CoLaus|PsyCoLaus***

Genome-wide genotyping was performed using the Affymetrix 500K SNP array. Nuclear DNA was extracted from whole blood of all participants.

Genotypes were called using BRLMM (http://www.affymetrix.com/support/technical/whitepapers/brlmm_whitepap). Duplicate individuals, and first and second degree relatives, were identified and then removed by computing estimates pair-wise genomic kinship coefficients, using KING ^1^. Subjects were excluded from the analysis in case of inconsistency between sex and genetic data, a genotype call rate of less than 90%, or inconsistencies of genotyping results in duplicate samples. Quality control for SNPs was performed using the following criteria: monomorphic (or with minor allele frequency (MAF) < 1%), call rates less than 90%, deviation from the Hardy-Weinberg equilibrium (HWE) (p < 1×10^-6^). Phased haplotypes were generated using SHAPEIT2 ^2, 3^. Imputation was performed using minimac3 ^4^ and the Haplotype Reference Consortium (HRC version r1.1) ^5^ hosted on the Michigan Imputation Server ^4^. We used imputed allele dosages for all SNPs to avoid genotyping missingness. A MAF > 1% and an imputation quality Rsq > 0.3 was required for the inclusion of the variant into further analyses. To account for possible population stratification, we computed principal component analysis (PCA) using PLINK ^6^ with default options. The first five ancestry-informative genetic principal components (PCs), gender and year of birth were used as fixed effects in the final analyses.

***1.2 NESDA|NTR***

Methods for biological sample collection and DNA extraction ^7^ and main quality control and imputation steps have been previously described ^8^. Briefly, genotyping was done on several platforms, with the large majority of samples genotyped on Affymetrix 6.0 Human SNP array. After platform-specific SNP quality control, missing SNP genotypes between platforms were cross-imputed using the GONL (Genomes of the Netherlands) ^9^ reference dataset. After stringent quality controls, a second round of imputations was then started to the 1000G Phase 3 all ancestries reference panel as available on the Michigan Imputation Server ^4^. The first five ancestry-informative genetic principal components (PCs), gender, year of birth were included as fixed effects in the final analyses.

***2. Mendelian Randomization (MR)***

***2.1 Selection of independent SNPs as instruments for MR***

The GWAS meta-analysis on BMI ^10^ identified 656 near-independent SNPs in 681,275 individuals whereas the GWAS meta-analysis on WHR ^11^ identified 370 independent SNPs in 694,649 individuals. Standard MR methods use instruments that are not in linkage disequilibrium (LD) with each other. To ensure this, we used the PLINK (version 1.9) ^6^ ‘clumping’ algorithm to select top associated SNPs and identify all SNPs in LD (r2 > 0.05) with the top associated SNP and ±5 Mb away in 489 European samples from 1000 Genomes Project phase 3 ^12^. P-value thresholds for “clumping” algorithm were set following the original manuscript recommendations to P < 1×10^−8^ for BMI and P < 5×10^−9^ for WHR, leaving respectively 610 and 339 genetic variants, available in both cohorts, for further MR analyses. An important assumption of MR is that each SNP must only influence risk of the outcome through the exposure under investigation, as the inclusion of SNPs that contribute through a pleiotropic pathway could bias estimates ^13^. To assess for the presence of directional horizontal pleiotropy, we used MR-Egger regression ^14^. We performed inverse variance weighted (IVW) instrumental variable analysis followed by sensitivity analyses based on weighted-median and simple-median causal estimators, which provide consistent causal estimates even when up to half of instruments are violating MR assumptions.

***2.2 MR analyses***

Analyses were conducted using the TwoSampleMR R package ^15, 16^. The “harmonise_data” function of TwoSampleMR R package excluded SNPs for being palindromic with intermediate allele frequencies. To further examine the robustness of our findings, appropriate analytical checks of heterogeneity were additionally performed by repeating IVW after excluding each of the SNPs, one at a time. Cochran’s (Q) statistic was calculated to provide an indication of heterogeneity between the estimates of the individual SNPs. For the CoLaus|PsyCoLaus and NESDA|NTR combined dataset variant rs747249 was dropped from analyses on WHR and the outcome non-atypical MDD vs controls, variant rs7591387 was dropped from the analyses on WHR and the outcome increase in appetite, and finally variant rs11919522 was dropped from the analyses on WHR and the outcome rejection sensitivity due to Cochran’s (Q) test.

**References**

1. Manichaikul A, Mychaleckyj JC, Rich SS, Daly K, Sale M, Chen WM. Robust relationship inference in genome-wide association studies. *Bioinformatics* 2010; **26**(22)**:** 2867-2873.

2. Delaneau O, Marchini J, Zagury JF. A linear complexity phasing method for thousands of genomes. *Nat Methods* 2011; **9**(2)**:** 179-181.

3. Delaneau O, Zagury JF, Marchini J. Improved whole-chromosome phasing for disease and population genetic studies. *Nat Methods* 2013; **10**(1)**:** 5-6.

4. Das S, Forer L, Schonherr S, Sidore C, Locke AE, Kwong A *et al.* Next-generation genotype imputation service and methods. *Nat Genet* 2016; **48**(10)**:** 1284-1287.

5. McCarthy S, Das S, Kretzschmar W, Delaneau O, Wood AR, Teumer A *et al.* A reference panel of 64,976 haplotypes for genotype imputation. *Nat Genet* 2016; **48**(10)**:** 1279-1283.

6. Purcell S, Neale B, Todd-Brown K, Thomas L, Ferreira MA, Bender D *et al.* PLINK: a tool set for whole-genome association and population-based linkage analyses. *Am J Hum Genet* 2007; **81**(3)**:** 559-575.

7. Boomsma DI, Willemsen G, Sullivan PF, Heutink P, Meijer P, Sondervan D *et al.* Genome-wide association of major depression: description of samples for the GAIN Major Depressive Disorder Study: NTR and NESDA biobank projects. *Eur J Hum Genet* 2008; **16**(3)**:** 335-342.

8. Mbarek H, Milaneschi Y, Hottenga JJ, Ligthart L, de Geus EJC, Ehli EA *et al.* Genome-Wide Significance for PCLO as a Gene for Major Depressive Disorder. *Twin Res Hum Genet* 2017; **20**(4)**:** 267-270.

9. Genome of the Netherlands C. Whole-genome sequence variation, population structure and demographic history of the Dutch population. *Nat Genet* 2014; **46**(8)**:** 818-825.

10. Yengo L, Sidorenko J, Kemper KE, Zheng Z, Wood AR, Weedon MN *et al.* Meta-analysis of genome-wide association studies for height and body mass index in approximately 700000 individuals of European ancestry. *Hum Mol Genet* 2018; **27**(20)**:** 3641-3649.

11. Pulit SL, Stoneman C, Morris AP, Wood AR, Glastonbury CA, Tyrrell J *et al.* Meta-analysis of genome-wide association studies for body fat distribution in 694 649 individuals of European ancestry. *Hum Mol Genet* 2019; **28**(1)**:** 166-174.

12. Genomes Project C, Auton A, Brooks LD, Durbin RM, Garrison EP, Kang HM *et al.* A global reference for human genetic variation. *Nature* 2015; **526**(7571)**:** 68-74.

13. Zheng J, Baird D, Borges MC, Bowden J, Hemani G, Haycock P *et al.* Recent Developments in Mendelian Randomization Studies. *Curr Epidemiol Rep* 2017; **4**(4)**:** 330-345.

14. Bowden J, Davey Smith G, Burgess S. Mendelian randomization with invalid instruments: effect estimation and bias detection through Egger regression. *Int J Epidemiol* 2015; **44**(2)**:** 512-525.

15. Hemani G, Tilling K, Davey Smith G. Orienting the causal relationship between imprecisely measured traits using GWAS summary data. *PLoS Genet* 2017; **13**(11)**:** e1007081.

16. Hemani G, Zheng J, Elsworth B, Wade KH, Haberland V, Baird D *et al.* The MR-Base platform supports systematic causal inference across the human phenome. *Elife* 2018; **7**.
